# Supplementary material for: N-Doped Carbon/CeO2 Composite as a Biomimetic Catalyst for Antibacterial Application
Source: Int J Mol Sci. 2023 Jan 26;24(3):2445. doi: 10.3390/ijms24032445 (PMC9916758; doi:10.3390/ijms24032445)
Supplement: Supplementary file 1 [file ijms-24-02445-s001.zip › ijms-2171443-supplementary.pdf]

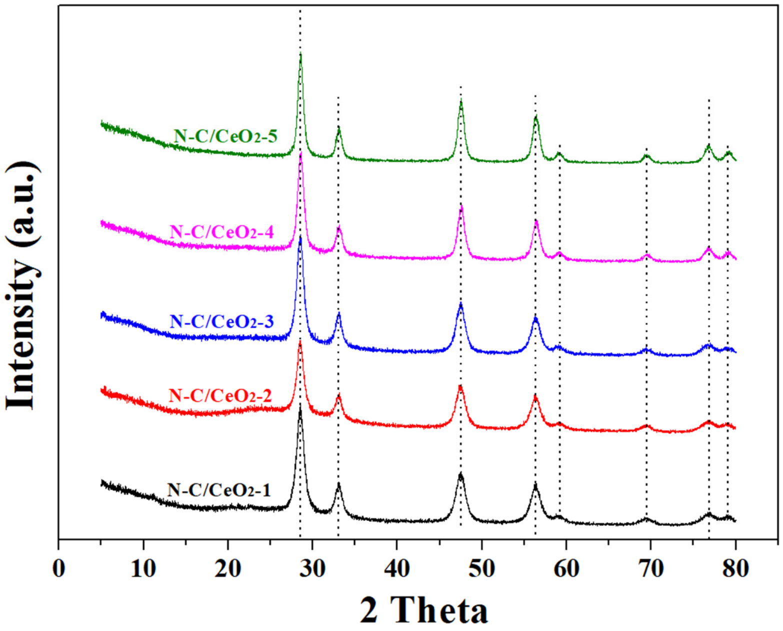

Figure S1. XRD pattern of the different N-C/CeO_2 composites. N-C/CeO_2-1, N-C/CeO_2-2, N-C/CeO_2-3, N-C/CeO_2-4 and N-C/CeO_2-5 were synthesized by using melamine and $Ce(NO_3)_3$•$6H_2O$ with the mass ratio of 6:1, 3:1, 2:1, 1:1 and 1:2, respectively.

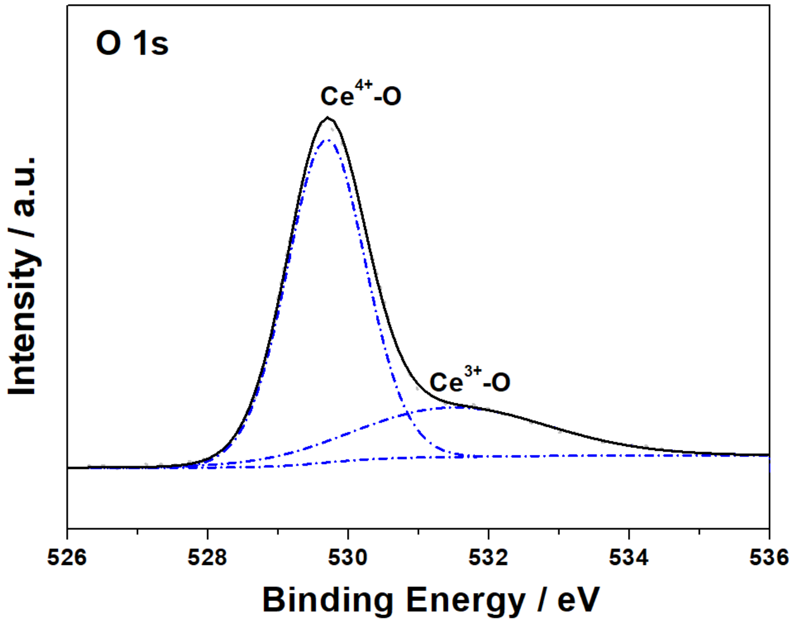

Figure S2. XPS pattern of N-C/CeO_2 composite O 1s.

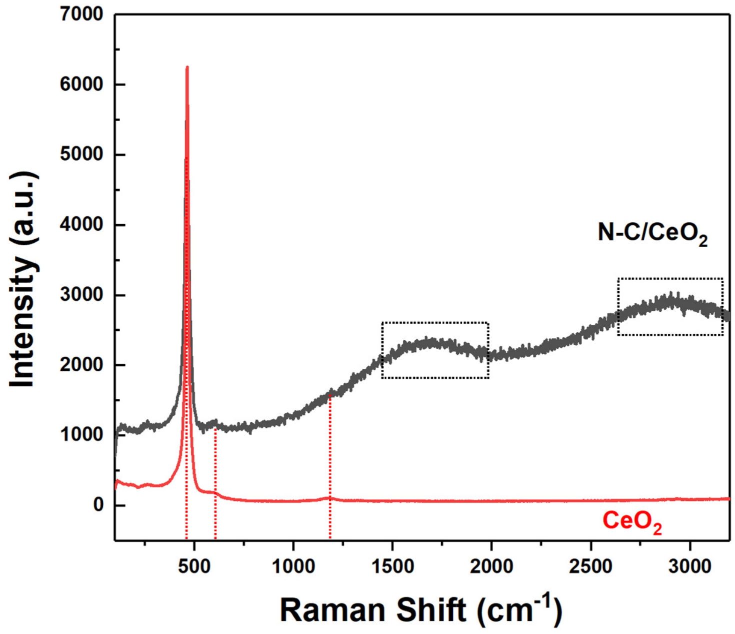

Figure S3. Raman spectra of CeO_2 and N-C/CeO_2 in the range 200~3200 cm^{-1}.

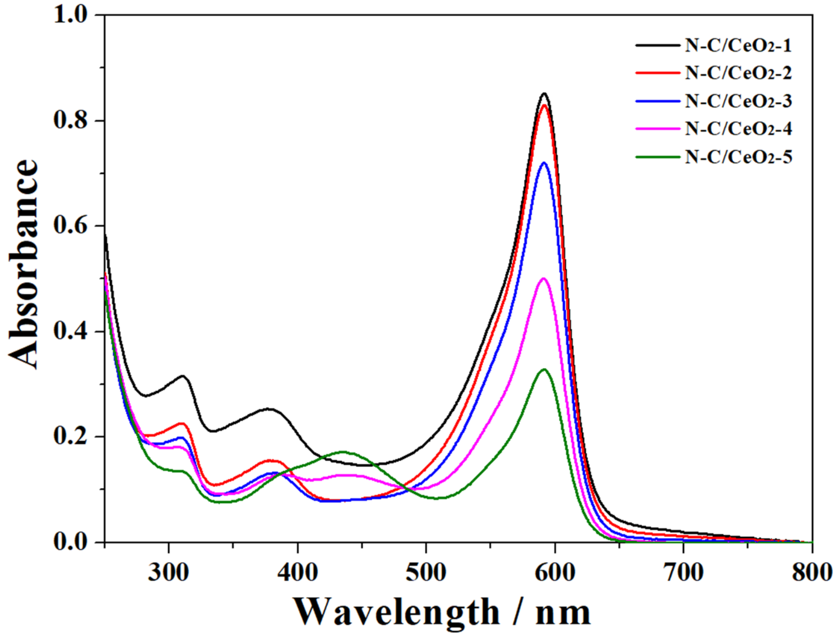

Figure S4. Absorbance spectra of reaction solutions catalyzed by the different N-C/CeO_2 composites.

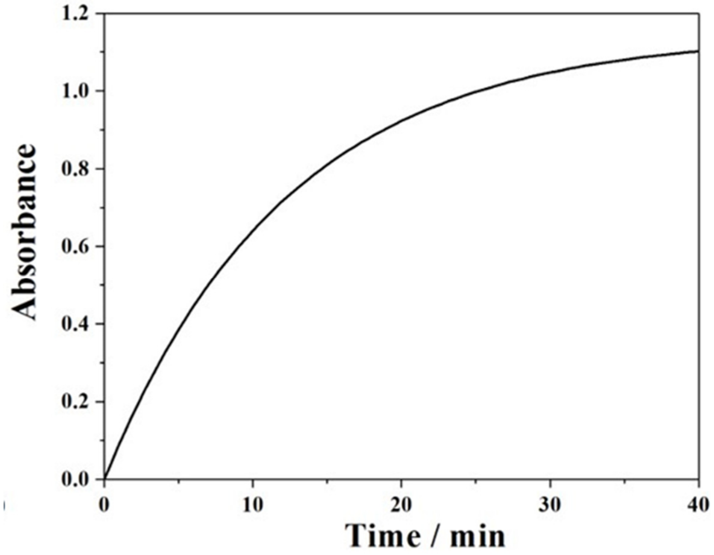

Figure S5. Time-dependent kinetics spectra showing the kinetics of the oxidative bromination of PR catalyzed by N-C/CeO_2 composite (40 min, 23–25 °C).

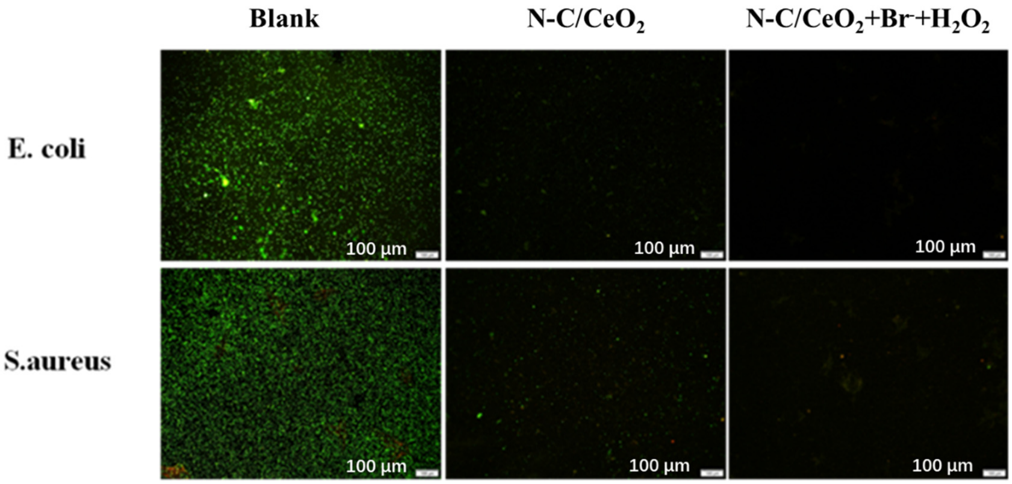

Figure S6. Live/dead staining images of *E. coli* and *S. aureus* treated with titanium plate in different systems: blank, N-C/CeO_2 and N-C/CeO_2 + Br^- + H_2O_2.

Table S1. The antibacterial of CeO_2 based materials.

Catalysts	Bacteria	Bacterial attachment (%)	Reference
CeO_{2-x} NRs	*E. coli*	~37	ACS Appl. Mater. Interfaces 2018, 10, 44722−44730
CeO_{2-x} nanorods with different aspect ratios	*E. coli*	~42%	ACS Sustainable Chemistry & Engineering 2020, 6744-6752
CeO_2@C	*E. coli*	~2.5%	Colloids and Surfaces A: Physicochemical and Engineering Aspects
N-C/CeO_2	*E. coli*	~0.24%	This work

References

1. Hu, M.; Korschelt, K.; Viel, M.; Wiesmann, N.; Kappl, M.; Brieger, J.; Landfester, K.; Therien-Aubin, H.; Tremel, W., Nanozymes in nanofibrous mats with haloperoxidase-like activity to combat biofouling. *ACS Applied Materials & Interfaces* **2018,** 10, (51), 44722-44730.
2. He, X.; Tian, F.; Chang, J.; Bai, X.; Yuan, C.; Wang, C.; Neville, A., Haloperoxidase mimicry by CeO_{2-x} nanorods of different aspect ratios for antibacterial performance. *ACS Sustainable Chemistry & Engineering* **2020,** 8, (17), 6744-6752.
3. Wang, N.; Li, W.; Ren, Y.; Duan, J.; Zhai, X.; Guan, F.; Wang, L.; Hou, B., Investigating the properties of nano core-shell CeO_2@C as haloperoxidase mimicry catalyst for antifouling applications. *Colloids and Surfaces A: Physicochemical and Engineering Aspects* **2021,** 608, 125592.
